# Matrix Metalloproteinases and Tissue Inhibitors of Metalloproteinases Are Potential Biomarkers of Pulmonary and Extra-Pulmonary Tuberculosis

**DOI:** 10.3389/fimmu.2020.00419

**Published:** 2020-03-11

**Authors:** Gokul Raj Kathamuthu, Nathella Pavan Kumar, Kadar Moideen, Dina Nair, Vaithilingam V. Banurekha, Rathinam Sridhar, Dhanaraj Baskaran, Subash Babu

**Affiliations:** ^1^National Institute for Research in Tuberculosis, National Institute of Health, International Center for Excellence in Research, Chennai, India; ^2^National Institute for Research in Tuberculosis (NIRT), Chennai, India; ^3^Government Stanley Medical Hospital, Chennai, India; ^4^Laboratory of Parasitic Diseases, National Institute of Allergy and Infectious Diseases, National Institutes of Health, Bethesda, MD, United States

**Keywords:** tuberculous lymphadenitis, MMPs, TIMPs, biomarkers, pulmonary tuberculosis

## Abstract

Matrix metalloproteinases (MMPs) and tissue inhibitors of metalloproteinase (TIMPs) are potential regulators of tuberculosis (TB) pathology. Whether they are candidates for non-sputum-based biomarkers for pulmonary TB (PTB) and extra-pulmonary TB (EPTB) is not fully understood. Hence, to examine the association of MMPs and TIMPs with PTB and EPTB, we have measured the circulating levels of MMPs (MMP-1, 2, 3, 7, 8, 9, 12, and 13) and TIMPs (TIMP-1, 2, 3, and 4) in PTB, EPTB and compared them with latent tuberculosis (LTB) or healthy control (HC) individuals. We have also assessed their circulating levels before and after the completion of anti-tuberculosis treatment (ATT). Our data describes that systemic levels of MMP-1, 8, 9, 12 were significantly increased in PTB compared to EPTB, LTB, and HC individuals. In contrast, MMP-7 was significantly reduced in PTB compared to EPTB individuals. Likewise, the systemic levels of MMP-1, 7, 13 were significantly increased in EPTB in comparison to LTB and HC individuals. In contrast, MMP-8 was significantly reduced in EPTB individuals compared to LTB and HC individuals. In addition, the systemic levels of TIMP-1, 2, 3 were significantly diminished and TIMP-4 levels were significantly enhanced in PTB compared to EPTB, LTB, and HC individuals. The circulating levels of TIMP-2 was significantly reduced and TIMP-3 was significantly elevated in EPTB individuals in comparison with LTB and HCs. Some of the MMPs (7, 8, 9, 12, 13 in PTB and 1, 7, 8, 9 in EPTB) and TIMPs (1, 2, 3, 4 in PTB and 4 in EPTB) were significantly modulated upon treatment completion. ROC analysis showed that MMP-1, 9 and TIMP-2, 4 could clearly discriminate PTB from EPTB, LTB and HCs and MMP-13 and TIMP-2 could clearly discriminate EPTB from LTB and HCs. Additionally, multivariate analysis also indicated that these alterations were independent of age and sex in PTB and EPTB individuals. Therefore, our data demonstrates that MMPs and TIMPs are potential candidates for non-sputum-based biomarkers for differentiating PTB and EPTB from LTB and HC individuals.

## Introduction

*Mycobacterium tuberculosis* (Mtb) kills nearly 1.5 million people globally and still poses a major threat with 90% of the disease occurring in developing countries (1, 2). Depending upon the Mtb exposure, infected individuals progress to a wide array of disease manifestations from symptomless latent TB (LTB) to active pulmonary TB (PTB) or extrapulmonary TB (EPTB) (3). Both PTB and EPTB suffer from diagnostic difficulties with low sensitivity of current diagnostic tests (4, 5). Most of the TB diagnostics depends upon the detection of pathogenic bacteria in sputum by culture, microscopy, or polymerase chain reaction (PCR) based assays like GeneXpert. However, difficulties do arise due to insufficient sputum collection, presence of few bacilli (paucibacillary) or extrapulmonary form of TB infection (6). Although the mortality rate of TB disease has reduced by 42% between the year 2000 and 2018, 3 million individuals are still undiagnosed or missed according to the World Health Organization (WHO) (7).

A systematic review in the year 2017 has shown that out of 399 biomarkers studied, only one urine-based biomarker (LAM, lipoarabinomannan) was considered as valid by WHO. However, LAM has minimal sensitivity [45%] and moderate specificity [56%] (8–10). Hence, it is essential to discover a rapid peripheral biomarker with a non-sputum test for diagnosis of pulmonary or extra-pulmonary TB and to distinguish between EPTB, PTB and LTB infected individuals (11, 12). Perhaps high-priority biomarkers with greater sensitivity ≥95% and specificity >75% to rule out or differentiate the disease status should be given as the second highest priority. Therefore, identification of new biomarkers should provide detailed information on disease pathogenesis with adequate predictive value for clinical use.

Matrix metalloproteinases (MMPs) are enzymes responsible for tissue destruction, disease spread and mortality (13). MMPs belongs to the class of membrane bound zinc-binding endopeptidases and are highly proficient in degrading the extracellular matrix and basement membrane (14, 15). Diverse forms of MMP have been characterized in vertebrates [twenty eight, 28 forms] and humans [twenty four, 24 forms], which carry out several essential functions. Tissue inhibitors of metalloproteinases (TIMPs) comprise a family of 4 homologous secreted (TIMP-1, 2, 3, 4) proteins (16). TIMPs are very important factors for TB disease, involved in tissue remodeling and repair upon destruction created by MMPs (17, 18). Previous studies have identified MMPs as markers of disease severity, bacterial burdens and as a biomarker for disease in PTB and EPTB (17, 19–21). Relatively, few studies have focused on examining the circulating levels of MMPs and TIMPs as immune biomarkers in both PTB and EPTB.

We show that the systemic levels of MMPs and TIMPs were different between PTB and EPTB disease compared to the other study (LTB and HC) groups. In addition, we have also observed significant discrimination among various MMPs (1, 9 for PTB and 13 for EPTB) and TIMPs (2, 4 for PTB, and 2 for EPTB) between the study groups upon ROC analysis. Therefore, we suggest that combinations of MMPs and TIMPs could be potential candidates for non-sputum-based biomarkers in discriminating PTB from EPTB and PTB and EPTB from LTB and HC individuals.

## Results

### Altered Circulating Levels of MMPs in PTB and EPTB Individuals

**Figure 1:**
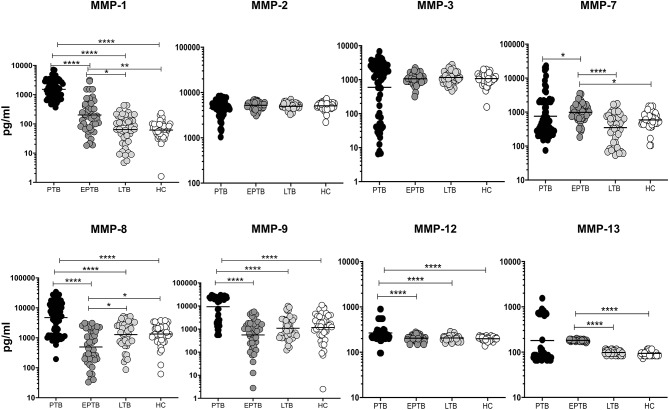
PTB and EPTB individuals exhibit altered circulating levels of matrix metalloproteinases (MMPs). The circulating levels of MMPs (1, 2, 3, 7, 8, 9, 12, 13) were examined in PTB (*n* = 68), EPTB (*n* = 44), LTB (*n* = 44) and in HC (*n* = 44) individuals. The results were given as scatter plots with each circle signifies a single individual and the bar representing the geometric mean and P values (*p < 0.05, **p < 0.01, ****p < 0.0001) were calculated using the Kruskal-Wallis test with Dunn's multiple comparisons.

We measured the circulating levels of MMPs (MMP-1, 2, 3, 7, 8, 9, 12, and 13) in PTB, EPTB, LTB, and HC individuals (Figure 1). The systemic levels of MMP-1 (geometric mean (GM) of PTB is 1522 pg/ml vs. GM of EPTB is 202.2 pg/ml vs. GM of LTB is 64.03 pg/ml and 61.36 pg/ml in HC), MMP-8 (GM of PTB is 4,722 pg/ml vs. GM of EPTB is 495.5 pg/ml vs. GM of LTB is 1,283 pg/ml and 1,342 pg/ml in HC), MMP-9 (GM of PTB is 9,270 pg/ml vs. GM of EPTB is 558.3 pg/ml vs. GM of LTB is 1,088 pg/ml and 1,171 pg/ml in HC) and MMP-12 (GM of PTB is 266.1 pg/ml vs. GM of EPTB is 204.7 pg/ml vs. GM of LTB is 206.6 pg/ml and 198.4 pg/ml in HC) were significantly higher in PTB individuals compared to EPTB, LTB, and HC individuals. In contrast, the circulating levels of MMP-7 (GM of PTB is 754.3 pg/ml vs. GM of EPTB is 987 pg/ml vs. GM of LTB is 348.6 pg/ml and GM of HC is 586.9 pg/ml) was significantly lower in PTB individuals compared to EPTB individuals.

As shown in Figure 1, the circulating levels of MMP-1 (GM of EPTB is 202.2 pg/ml vs. 64.03 pg/ml in LTB and 61.36 pg/ml in HC), MMP-7 (GM of EPTB is 987 pg/ml vs. 348.6 pg/ml in LTB and 586.9 pg/ml in HC) and MMP-13 (GM of EPTB is 182.3 pg/ml vs. 97.97 pg/ml in LTB and 94.21 pg/ml in HC) were significantly higher in EPTB individuals when compared to LTB and HC individuals. In contrast, the circulating levels of MMP-8 (GM of EPTB is 495.5 pg/ml vs. 1,283 pg/ml in LTB and 1,342 pg/ml in HC) was significantly lower in EPTB compared to LTB and HC individuals. Thus, both PTB and EPTB are associated with altered plasma levels of MMPs.

### Altered Circulating Levels of TIMPs in PTB and EPTB Individuals

**Figure 2:**
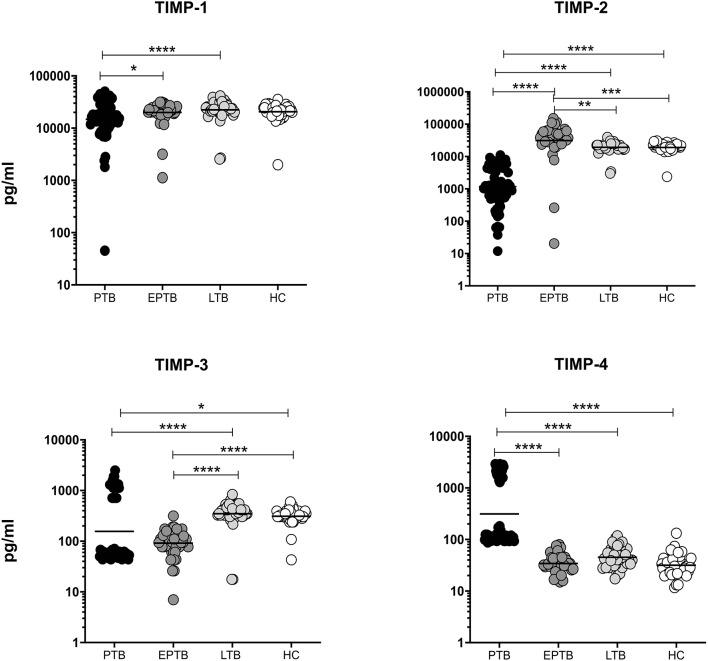
PTB and EPTB individuals are associated with altered plasma levels of TIMPs. The systemic levels of TIMPs (1, 2, 3, 4) were examined in PTB (*n* = 68), EPTB (*n* = 44), LTB (*n* = 44), and in HC (*n* = 44) individuals. The results were given as scatter plots with each circle indicates a single individual and the bar representing the geometric mean. P values (*p < 0.05, **p < 0.01, ***p < 0.001, ****p < 0.0001) were calculated using the Kruskal-Wallis test with Dunn's multiple comparisons.

We measured the circulating levels of TIMPs (TIMP-1, 2, 3, 4) in PTB, EPTB, LTB, and HC individuals. As shown in Figure 2, TIMP-1 (GM of PTB is 14,720 pg/ml vs. 19,959 pg/ml in EPTB vs. 22,462 pg/ml in LTB and 20,596 pg/ml in HC) and TIMP-2 (GM of PTB is 1,173 pg/ml, GM of EPTB is 31,037 pg/ml vs. 19,249 pg/ml in LTB and 19,129 pg/ml in HC) and TIMP-3 (GM of PTB is 156.6 pg/ml GM of EPTB is 91.71 pg/ml vs. 350.4 pg/ml in LTB and 312.2 pg/ml in HC) and levels were significantly lower in PTB individuals compared to EPTB, LTB and HC individuals. In contrast, TIMP-4 levels were significantly higher in PTB (GM of PTB is 312.4 pg/ml GM of EPTB is 34.19 pg/ml vs. 45.06 pg/ml in LTB and 31.69 pg/ml in HC) compared to EPTB, LTB, and HC individuals.

The circulating levels of TIMP-2 (GM of EPTB is 31,037 pg/ml vs. 19,249 pg/ml in LTB and 19,129 pg/ml in HC) was significantly higher in EPTB individuals in comparison with LTB and HC individuals. In contrast, the systemic levels of TIMP-3 (GM of EPTB is 91.71 pg/ml vs. 350.4 pg/ml in LTB and 312.2 pg/ml in HC) was significantly lower in EPTB individuals in comparison with LTB and HC individuals (Figure 2). Hence, both PTB and EPTB are associated with altered circulating levels of TIMPs.

### Post-treatment Modulation of MMPs in PTB Individuals

**Figure 3:**
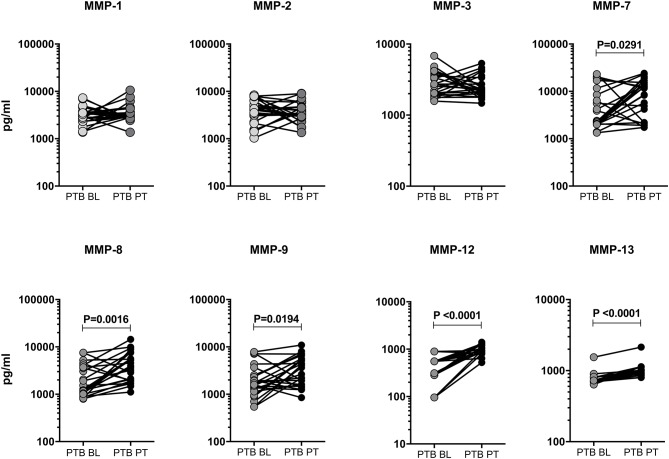
Circulating levels of MMPs were modulated upon completion of ATT in PTB individuals. The plasma levels of MMPs (1, 2, 3, 7, 8, 9, 12, 13) were measured in a subset of PTB individuals (*n* = 24) before (baseline, BL) and after the completion of 6 months (post-treatment, PT) of ATT. The data were represented as line bars with each line representing a single individual and P values were calculated using the Wilcoxon signed rank test.

We measured the pre and post-treatment circulating levels of MMPs in a subset of PTB individuals (Figure 3). As shown in Figure 3, the systemic levels of MMP-7 [3855.0 pg/ml in BL vs. 7653.0 pg/ml in post-T], MMP-8 [1833.0 pg/ml in BL vs. 3734.0 pg/ml in post-T], MMP-9 [1849.0 pg/ml in BL vs. 3060.0 pg/ml in post-T], MMP-12 [353.0 pg/ml in BL vs. 964.2 pg/ml in post-T] and MMP-13 [765.8 pg/ml in BL vs. 975.1 pg/ml in post-T] were significantly increased in post-treatment condition than with pre-treatment levels among PTB individuals. However, the other MMPs (1, 2, 3) were not significantly different between the two time points. Thus, PTB individuals are associated with increase of certain MMPs after ATT.

### Post-treatment Modulation of MMPs in EPTB Individuals

**Figure 4:**
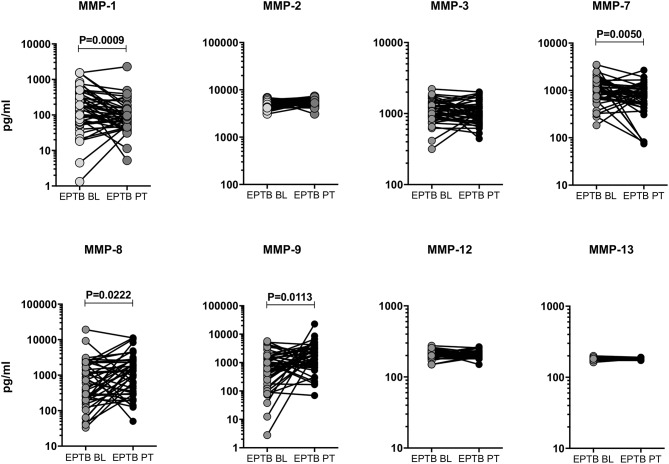
Systemic levels of MMPs were modulated after ATT in EPTB individuals. The circulating levels of MMPs (1, 2, 3, 7, 8, 9, 12, 13) were examined in EPTB individuals (*n* = 44) before (baseline, BL) and after the completion of 6 months (post-treatment, PT) of ATT. The data were represented as line bars with each line representing a single individual and P values were calculated using the Wilcoxon signed rank test.

To study the effect of ATT, we have measured the baseline (BL) and post-treatment (post-T) systemic levels of MMPs (MMP-1, 2, 3, 7, 8, 9, 12, and 13) in EPTB individuals (Figure 4). As we shown in Figure 4, the circulating levels of MMPs were significantly (MMP-1 [146.2 pg/ml in BL vs. 87.82 pg/ml in post-T] and MMP-7 [987.0 pg/ml in BL vs. 87.82 pg/ml in post-T] was diminished) and (MMP-8 [575.5 pg/ml in BL vs. 1052.0 pg/ml in post-T] and MMP-9 [558.0 pg/ml in BL vs. 1512.0 pg/ml in post-T] was increased) altered between baseline and post-treatment condition of EPTB individuals. In contrast, the circulating levels of other MMPs (MMP-2, 3, 12, and 13) were not significantly modulated upon the completion of ATT.

### Post-treatment Modulation of TIMPs in PTB and EPTB Individuals

**Figure 5:**
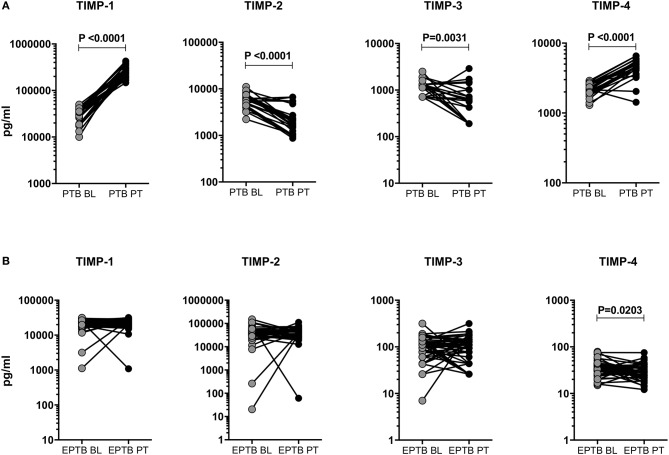
Post-treatment modulation of TIMPs upon completion of ATT in PTB and EPTB individuals. The systemic levels of TIMPs (1, 2, 3, 4) were measured in PTB **(A)** and EPTB **(B)** individuals before (baseline, BL) and 6 months (post-treatment, PT) after the completion of ATT. The data were represented as line bars with each line representing a single individual and P values were calculated using the Wilcoxon signed rank test.

Like MMPs, we also wanted to study the effect of ATT in TIMPs and examined the pre and post-treatment systemic levels of TIMP-1, 2, 3, 4 in PTB and EPTB individuals (Figure 5). As shown in Figure 5A, in PTB individuals, the circulating levels of TIMP-1 (28440.0 pg/ml in BL vs. 254551.0 pg/ml in post-T) and TIMP-4 (2188.0 pg/ml in BL vs. 4364.0 pg/ml in post-T) were significantly increased at post-treatment compared to pre-treatment levels. In contrast, the systemic levels of TIMP-2 (5546.0 pg/ml in BL vs. 2107.0 pg/ml in post-T) and TIMP-3 (1141.0 pg/ml in BL vs. 555.5 pg/ml in post-T) were significantly diminished at post-treatment compared to pre-treatment levels (Figure 5A). The circulating levels of TIMP-4 (34.19 pg/ml in BL vs. 28.42 pg/ml in post-T) was significantly decreased between baseline and post-treatment condition of EPTB individuals. In contrast, the systemic levels of TIMP-1, 2, 3 were not significantly altered between the baseline and post-treatment condition of EPTB individuals (Figure 5B).

### MMPs (1, 9) and TIMPs (2, 4) Distinguish PTB From EPTB, LTB, and/or HC Individuals

To elucidate whether MMPs and TIMPs can distinguish PTB from other groups, we performed ROC analysis (Table 2). Among the MMPs analyzed, MMP-1 (PTB vs. EPTB-sensitivity-95.59, specificity-84.09, AUC-0.9418 and *P* < 0.0001; PTB vs. LTB-sensitivity-98.53, specificity-100, AUC-0.9993 and *P* < 0.0001; PTB vs. HC-sensitivity-100, specificity-100, AUC-1 and *P* < 0.0001) and MMP-9 (PTB vs. EPTB-sensitivity-80.88, specificity-84.09, AUC-0.9094 and *P* < 0.0001; PTB vs. LTB- sensitivity-73.53, specificity-75.00, AUC-0.8763, and *P* < 0.0001; PTB vs. HC-sensitivity-100, specificity-100, AUC-1 and *P* < 0.0001) could significantly discriminate between the study individuals. Similarly, TIMP-2 (PTB vs. EPTB-sensitivity-95.59, specificity-95.45, AUC-0.9576 and *P* < 0.0001; PTB vs. LTB-sensitivity-100, specificity-95.45, AUC-0.9853 and *P* < 0.0001; PTB vs. HC-sensitivity-100, specificity-97.73, AUC-0.9923, and *P* < 0.0001) and TIMP-4 (PTB vs. EPTB-sensitivity-100, specificity-100, AUC-1 and *P* < 0.0001; PTB vs. LTB-sensitivity-98.53, specificity-95.45, AUC-0.9789 and *P* < 0.0001; PTB vs. HC-sensitivity-100, specificity-97.73, AUC-0.9866 and *P* < 0.0001) could significantly distinguish PTB from other study groups (Table 2). Hence, MMP-1, 9 and TIMP-2, 4 are potential markers in distinguishing PTB from EPTB, LTB, and HC groups.

### MMP-13 and TIMP-2 Discriminate EPTB From LTB and HC Individuals

Similar to PTB, the ROC analysis of MMPs and TIMPs was carried out between EPTB, LTB, and HC individuals (Table 3). Among the MMPs analyzed, MMP-13 (EPTB vs. LTB-sensitivity-100, specificity-100, AUC-1, and *P* < 0.0001; EPTB vs. HC-sensitivity-100, specificity-100, AUC-1 and *P* < 0.0001) could significantly discriminate EPTB from LTB and HC individuals. As shown in Table 3, the systemic levels of TIMP-2 could significantly distinguish EPTB from LTB (sensitivity-81.82, specificity-90.91, AUC-0.8631, and *P* < 0.0001) and HC (sensitivity-84.09, specificity-84.09, AUC-0.8719, and *P* < 0.0001) individuals. Thus, MMP-13 and TIMP-2 are potential candidate markers for distinguishing EPTB from LTB and HC individuals.

### Relationship Between MMPs/TIMPs in Different TB Infected and HC Individuals

**Figure 6:**
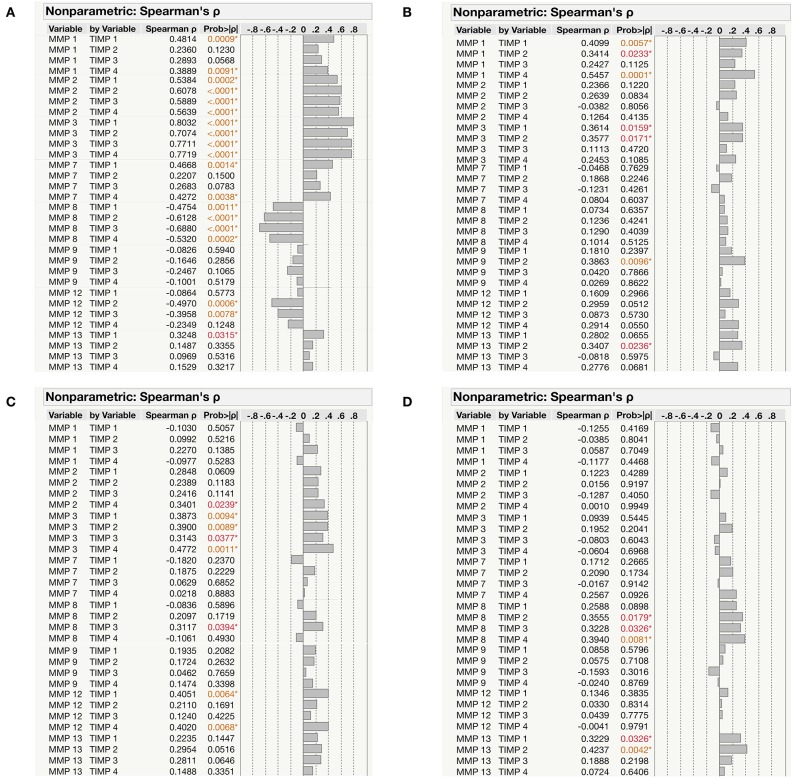
Correlation between the MMPs (1, 2, 3, 7, 8, 9, 12, 13) and TIMPs (1, 2, 3, 4) levels among the study individuals **(A)** PTB **(B)** EPTB **(C)** LTB and **(D)** HCs. The data were represented as table and the significant differences were given in orange or red color.

We next examined the correlation of MMPs (1, 2, 3, 7, 8, 9, 12, 13) with TIMPs (1, 2, 3, 4) in the different group of (PTB, EPTB, LTB, and HC) individuals. As shown in Figure 6, MMP-2 and MMP-3 were positively correlated and MMP-8 was negative correlated with all TIMPs (*P* < 0.0001); whereas, a significant positive correlation was observed for MMP-1, MMP-7, and MMP-13 with TIMP-1 and/or TIMP-4 in PTB individuals. In contrast, MMP-12 exhibited a significant negative correlation with TIMP-2 and TIMP-3 in PTB individuals (Figure 6A). Similarly, MMP-1 and MMP-3 were positively correlated with TIMP-1 and TIMP-2, whereas MMP-9 and MMP-13 were positively correlated with TIMP-2 alone in EPTB individuals (Figure 6B). As shown in Figure 6C, MMP-3 levels were positively correlated with all TIMPs; whereas, MMP-2, MMP-8, and MMP-12 were positive correlated with TIMP-4/TIMP-3/TIMP-1 and TIMP4, respectively, in LTB individuals. Finally, the plasma levels of MMP-8 and MMP-13 were shown to be positively correlated with certain TIMPs (2, 3, 4) and (1, 2) in HC individuals (Figure 6D). Thus, based on our observation certain MMPs appears to be significantly correlated with TIMPs.

### PCA Analysis of MMPs/TIMPs Between the Study Individuals

**Figure 7:**
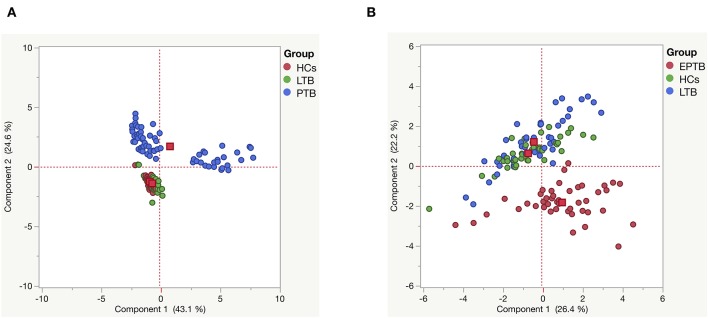
Principle component analysis (PCA) plots of MMPs (1, 2, 3, 7, 8, 9, 12, 13) and TIMPs (1, 2, 3, 4) **(A)** PTB (blue) vs. LTB (green) vs. HC (red) and **(B)** EPTB (red) vs. LTB (blue) vs. HC (green) individuals. PCA plot shows the ELISA data from the combination of three different experimental groups.

Next, we analyzed the impact of MMPs (1, 2, 3, 7, 8, 9, 12, 13) and TIMPs (1, 2, 3, 4) in discriminating the diseased individuals from infected and uninfected individuals by PCA analysis. We have utilized their plasma levels to generate the clusters for PTB and EPTB individuals and compared them with LTB and HC individuals (Figure 7). We observed distinct clustering of MMPs/TIMPs for both PTB (component 1–43.1% and component 2–24.6) and EPTB individuals (component 1–26.4% and component 2–22.2) which are able to clearly discriminate from LTB and HC individuals (Figures 7A,B).

### Logistic Regression Analysis of PTB and EPTB

Finally, we have performed univariate and multivariate analysis (95% confidence interval [CI]) of MMPs (1, 2, 3, 7, 8, 9, 12, 13) and TIMPs (1, 2, 3, 4) in both PTB and EPTB after the adjustment for confounding factors like age and gender to identify whether they might possibly serve as biomarkers for PTB and EPTB disease. As shown in Table 4, we found that by both univariate and multivariate analysis, MMP-1, 7, 8, 9, 12, and 13 were associated with significantly greater odds of PTB, while MMP-2 and 3 were associated with significantly decreased odd of PTB. Similarly, TIMP-4 was associated with highly elevated odd of PTB, while TIMP-1 and 2 were associated with decreased odds of PTB. As shown in Table 5, we also found that by both univariate and multivariate analysis, MMP-2, TIMP-1, 2, 3 were associated with significantly greater odds of EPTB while MMP-1, 7, 9, 13 and TIMP-4 were associated with significantly decreased odd of EPTB. Thus, logistic regression analysis identified the odds ratio of MMP and TIMP levels being associated with increased or decreased likelihood of having PTB or EPTB.

## Discussion

The identification of definitive non-sputum, blood-based biomarkers for PTB and EPTB is essential either for disease diagnosis or for chemotherapy treatment monitoring. It is also worthwhile to understand the architecture and mechanism of disease pathogenesis which might provide additional targets for immune mediated therapies. The role of certain MMPs and TIMPs were studied previously in the immunopathology of pulmonary TB infection and certain forms of EPTB. Nevertheless, a comprehensive examination of a complete panel of MMPs and TIMPs in PTB and EPTB has not been performed till now. Hence in the present study, we have examined the association of MMPs and TIMPs in two different forms of TB and compared them with LTB and HCs. The systemic levels of both MMPs and TIMPs disclose that an alteration occurred between the PTB and EPTB patients indicating their disease presentation profile differs significantly. ROC analysis discriminated between the diseased and the other study groups among some of the MMPs and TIMPs suggesting that they have the potential ability to be used as a blood-based biomarker.

Several studies have revealed that PTB individuals are characterized by enhanced MMPs (MMP-1, 2, 3, 7, 8, 9) in sputum, pleural fluid and bronchoalveolar lavage (BAL) fluids (17, 22–25). Our data were also consistent with the above findings and we show that the circulating levels of MMPs (1, 8, 9, 12) and MMPs (1, 7, 13) were significantly increased in both PTB and EPTB individuals. Enhanced systemic levels of MMP-1 in both TB diseased groups supports the premise that it is an absolute indicator of tissue matrix damage, higher alveolar destruction and breakdown of collagen. Another study has also revealed that MMP-1 triggers the lung matrix destruction and their levels were increased compared to latently infected individuals (26). Even microarray profiling has shown increased (660 fold) MMP-1 gene expression in human TB granulomas, cavity areas (rabbit model) and macaque lungs compared to healthy lungs (27–29). Previous study also highlighted similar data indicating elevated MMP-1 and HO-1 levels were highly discriminatory between active TB and LTB individuals (30). It has been observed that increased levels of MMPs (2, 8, 9) at diagnosis and higher MMP-3 and 8 at 2 weeks were connected to culture positivity in sputum samples at 2-weeks of infection. After the first 6 weeks of treatment initiation, both MMP-1 and 8 levels remains high with delayed sputum culture conversion (31). We also describe similar post-treatment data on MMP-8 where the circulating levels are higher after treatment and MMP-1 levels were not significantly altered after chemotherapy.

In addition, systemic levels of MMPs (1, 7, 8) were significantly increased in children with active TB than healthy individuals (32). Elevated sputum and plasma levels of MMP-8 are present in TB individuals, TB- immune reconstitution inflammatory syndrome (IRIS) and in individuals with and without HIV co-infection (33–35). MMP-9 levels were correlated with disease severity and increases the susceptibility TB infection (36). The levels of MMP-9 were elevated in the cerebrospinal fluid of TB meningitis patients and pleural fluid. Elevated levels of MMP-12 were observed in the COPD patient than the control groups (37). Higher secretion of MMP-9 as found upon Mtb infection of both monocytes and macrophages, and was shown to be important for granuloma formation (38, 39). It also been shown that MMP-9 gene knockout mice have poor granuloma architecture and reduced recruitment of macrophages (36). Similarly, increased MMP-8 levels in PTB but not EPTB implies that this neutrophil-derived MMP could be related to severe form of TB disease (40). Likewise, our data were similar to the above findings by showing increased levels of MMP-8, MMP-9, and MMP-12 in PTB compared EPTB, LTB and HC groups. Their increased levels might be either deleterious to the host or important for the maintenance of the active granuloma. Even the post treatment systemic levels of MMP-9, 12, 13 were higher compared to pre-treatment indicating MMP levels could serve as additional biomarkers for successful chemotherapy. In addition, the ROC analysis revealed certain MMPs (1, 9) and MMP-12 (PTB vs. HC) were potentially capable of being a good peripheral bio-markers for PTB to separate from other diseased or control individuals.

In contrast, MMP-8 were significantly diminished in EPTB compared to LTB and HC groups. The reason could be because of different site of infection between the two TB infected groups. Hence, both diseased groups differ significantly on their expression levels stating that it might be used as a peripheral based diagnostic bio-marker for EPTB disease. After the completion of ATT, the systemic levels of MMP-8 were significantly downregulated in EPTB individuals. Our data also shows higher circulating levels of MMP-7 and MMP-13 in EPTB individuals compared to PTB, LTB and/or HC individuals. The post-treatment circulating levels of MMP-7 was significantly decreased compared to pre-treatment levels. MMP-13 could potentially be of use as a blood-based bio-marker for diagnosis of EPTB disease. Consistent with our data, the mRNA expression levels of certain MMPs (1, 3, 12, 13) were highly upregulated in macrophages or epithelial tissues isolated from infected tissues (41).

It has been implied that Mtb dynamically impairs the equilibrium between MMPs and TIMPs. Moreover, whether TIMPs could be used as a potential immune based biomarker in PTB and EPTB remains unclear (29). In our study, the systemic levels of TIMP-1 were significantly reduced in both TB diseased groups. Similar to our observation, lower TIMP-1 levels were reported in pulmonary secretions of TB patients (42). It has also been shown in cell culture experiments, the elevated levels of MMP is independent and not balanced by a higher TIMP-1 level (43). We also observed circulating TIMP-1 levels were decreased in active TB patients but not in EPTB individuals. We predict that lower TIMP-1 levels in PTB could be due to unrestricted gelatinolytic action within the granuloma architecture with a subsequent propensity for matrix degradation. ROC analysis of TIMP-1 potentially discriminated PTB from EPTB and other control groups. Hence, we propose that TIMP-1 could be a better biomarker for active TB diagnosis alone.

Similar to TIMP-1, TIMP-2 plasma levels were also significantly diminished in PTB and elevated in EPTB compared to LTB and HC individuals. Hence, it might be used as a potential biomarker for discriminating between PTB and EPTB disease and also from LTB and HC individuals. This is in contrast to some other studies where they displayed significantly higher TIMP-1 and TIMP-2 levels in TB cases compared to healthy controls (17, 42). The reason behind this difference observed between the various data are yet to be explored. We have also shown that the circulating levels of TIMP-3 were significantly decreased in both the diseased groups. Previous study has revealed that TIMP-3 was greatly decreased in *in-vitro* human monocyte infection models but not at the tissue site (43). There was also evidence from mice models showing the reduction in TIMP-3 levels was connected with degradation of extra cellular matrix (44). Finally, our data on TIMP-4 revealed an increase in PTB compared to EPTB, LTB and HC individuals. In PTB, the systemic levels of TIMPs (TIMP-1, 4 were increased and TIMP-2, 3 were diminished) were altered after the completion of chemotherapy. In contrast, TIMP-4 alone was significantly modulated after the treatment in EPTB individuals. Finally, it was clearly seen from the ROC data of TIMPs where PTB (TIMP-2 and TIMP-4) and EPTB (TIMP-2) were highly discriminated from LTB and HC individuals. Hence, the above mentioned TIMPs might be a very good blood-based biomarker for PTB and EPTB diagnosis.

Our PCA analysis reveals that MMPs and TIMPs as a whole are useful parameters to distinguish PTB from LTB and HC individuals and EPTB from LTB and HC individuals with minimal overlapping distributions. This greatly adds to the growing evidence in the literature about the importance of MMPs and TIMPs are potential biomarkers of PTB and EPTB. Moreover, our data also provide additional insight into the equilibrium between MMPs and TIMPs in the different study groups by comparing the correlation matrices of these biomarkers. While, we observe a mostly positive correlation in EPTB, we observe a mix of positive and negative associations in PTB with MMP/TIMP correlations. Finally, our data also reveal the associations of MMPs and TIMPs with either increased or decreased risk of PTB and EPTB with certain MMPs and TIMPs being clearly positively associated and other being negatively associated with pulmonary or extra-pulmonary disease. This is therefore an important value addition tool to the armamentarium of biomarkers reflecting these disease processes.

The limitations of the study include the moderate sample size, the lack of a validation cohort, the inclusion of only one form of EPTB and the absence of other bacterial, viral or parasitic infections. Overall from our observation, we suggest that differences in the systemic levels of various MMPs and TIMPs can be utilized as potential blood-based biomarkers for TB disease. Since, obtaining either sputum or bronchoalveolar lavage (BAL) fluids is challenging, this if validated in larger studies should provide a surrogate marker for these conditions.

## Materials and Methods

### Study Population

The present study was approved by Institutional Review Board (NIRTIEC2010007) of National Institute for Research in Tuberculosis (NIRT), Chetpet, Chennai, Tamil Nadu, India and informed written consent form was acquired from all the study individuals. Our study consists of four different [PTB (*n* = 68), EPTB (*n* = 44), latent TB [LTB] (*n* = 44), and healthy controls [HC] (*n* = 44)] groups. The demographics of the study population are given in Table 1. PTB was diagnosed on the basis of culture positivity for Mtb by solid culture. EPTB diseased patients had only cervical lymphadenopathy and were diagnosed based on histopathology or bacteriological investigation comprising of GeneXpert or culture positive for Mtb. LTB individuals were positive for QuantiFERON TB-Gold (QFT) in tube assay and had lack of abnormalities in chest radiography and absence of any pulmonary symptoms. HCs were QFT negative and had lack of abnormalities in chest radiography and absence of any pulmonary symptoms. All the study individuals were HIV negative and devoid of steroid treatment and not affected with other chronic viral or bacterial infection. Plasma samples were collected at baseline (pre-treatment) from all the four groups of individuals. Both PTB and EPTB individuals were administered standard anti-tuberculosis treatment for 6 months and fresh plasma samples were collected from a subset of PTB (*n* = 24) and all EPTB (*n* = 44) individuals at the end of treatment.

**Table 1:** Demographics of the study individuals.

**Study demographics**	**PTB**	**EPTB**	**LTB**	**HC**
Number of subjects recruited *(n)*	68	44	44	44
Gender (M/F)	41/27	26/18	29/15	24/20
Median age in years (Range)	31 (19–54)	30 (18–51)	32 (21–62)	34 (21–55)
Culture/smear grade (0/1+ /2+ /3+)	0/23/27/18	8/34/2/0	Not done	Not done
QuantiFERON-TB Gold	Not done	Not done	Positive	Negative

**Table 2:** MMPs (1, 9) and TIMPs (2, 4) clearly distinguish PTB from EPTB, LTB, and/or HC individuals.

	**PTB vs. EPTB**				**PTB vs. LTB**				**PTB vs. HC**			
	**Sensitivity**	**Specificity**	***P***	**AUC**	**Sensitivity**	**Specificity**	***P***	**AUC**	**Sensitivity**	**Specificity**	***P***	**AUC**
**MMP-1**	**95.59**	**84.09**	**<0.0001**	**0.9418**	**98.53**	**100**	**<0.0001**	**0.9993**	**100**	**100**	**<0.0001**	**1.000**
MMP-2	61.76	61.36	0.0965	0.5932	52.94	50.00	0.6723	0.5237	57.35	52.27	0.2577	0.5635
MMP-3	57.35	56.82	0.2730	0.5615	57.35	54.55	0.5238	0.5358	57.35	54.55	0.3055	0.5575
MMP-7	61.76	72.73	0.0347	0.6185	50.00	50.00	0.0410	0.6146	57.35	61.36	0.2836	0.5602
MMP-8	72.06	72.73	<0.0001	0.8496	66.18	77.27	<0.0001	0.7988	67.65	70.45	<0.0001	0.7741
**MMP-9**	**80.88**	**84.09**	**<0.0001**	**0.9094**	**73.53**	**75.00**	**<0.0001**	**0.8763**	**72.06**	**79.55**	**<0.0001**	**0.8469**
MMP-12	74.00	65.91	<0.0001	0.7694	63.24	75.00	<0.0001	0.7602	**75.00**	**81.82**	**<0.0001**	**0.8319**
MMP-13	63.24	100	0.0156	0.6357	51.47	81.82	0.6464	0.5257	48.53	75.00	0.7658	0.5167
TIMP-1	69.12	77.27	0.0056	0.6554	70.59	84.09	0.0004	0.6999	69.12	68.18	0.0042	0.6608
**TIMP-2**	**95.59**	**95.45**	**<0.0001**	**0.9576**	**100**	**95.45**	**<0.0001**	**0.9853**	**100**	**97.73**	**<0.0001**	**0.9923**
TIMP-3	81.82	84.09	<0.0001	0.8285	64.71	95.45	0.0325	0.6200	64.71	97.73	0.0183	0.6324
**TIMP-4**	**100**	**100**	**<0.0001**	**1.0000**	**98.53**	**95.45**	**<0.0001**	**0.9789**	**100**	**97.73**	**<0.0001**	**0.9866**

*ROC analysis was performed to determine the sensitivity, specificity, and area under the curve using the plasma levels of MMPs (1, 2, 3, 7, 8, 9, 12, 13) and TIMPs (1, 2, 3, 4) between PTB vs. EPTB, PTB vs. LTB and PTB vs. HC to calculate the ability of these factors to distinguish PTB from EPTB, LTB and HC individuals. Highest discrimination were represented in bold*.

**Table 3:** MMP-13 and TIMP-2 clearly distinguishes EPTB from LTB and HC individuals.

	**EPTB vs. LTB**				**EPTB vs. HC**			
	**Sensitivity**	**Specificity**	***P***	**AUC**	**Sensitivity**	**Specificity**	***P***	**AUC**
MMP-1	59.09	70.45	0.0037	0.6798	75.00	63.64	<0.0001	0.7433
MMP-2	61.36	65.19	0.0489	0.6219	59.09	54.55	0.5579	0.3500
MMP-3	56.82	54.55	0.3247	0.5610	54.55	52.77	0.7011	0.5238
MMP-7	75.00	70.45	<0.0001	0.8011	75.00	61.36	0.0003	0.7237
MMP-8	65.91	54.55	0.0099	0.6596	61.36	70.45	0.0026	0.6865
MMP-9	50.00	54.55	0.1186	0.5966	54.55	65.91	0.0159	0.6493
MMP-12	52.27	54.55	0.9800	0.5015	52.27	61.36	0.1465	0.5889
**MMP-13**	**100**	**100**	**<0.0001**	**1**	**100**	**100**	**<0.0001**	**1**
TIMP-1	56.82	63.64	0.0639	0.6147	59.09	52.27	0.4678	0.5449
**TIMP-2**	**81.82**	**90.91**	**<0.0001**	**0.8631**	**84.09**	**84.09**	**<0.0001**	**0.8719**
**TIMP-3**	64.71	81.82	0.0344	0.6186	**97.73**	**95.45**	**<0.0001**	**0.9587**
TIMP-4	61.36	72.73	0.0022	0.6896	68.18	52.27	0.2817	0.5666

*ROC analysis was performed to determine the sensitivity, specificity, and area under the curve using the plasma levels of MMPs (1, 2, 3, 7, 8, 9, 12, 13) and TIMPs (1, 2, 3, 4) between EPTB vs. LTB and EPTB vs. HC individuals to calculate the ability of these factors to distinguish EPTB from LTB and HC individuals. Highest discrimination were represented in bold*.

**Table 4:** Logistic regression model to identify the biomarkers for PTB.

	**Univariate**		**Multivariate**	
	**OR (95% CI)**	***P***	aOR (95% CI)^*^	***P***
MMP1	9.15 (4.28–19.56)	<0.001	10.34 (3.31–32.27)	<0.001
MMP2	0.64 (0.52–0.77)	<0.001	0.65 (0.53–0.79)	<0.001
MMP3	0.76 (0.64–0.9)	0.002	0.75 (0.62–0.92)	0.005
MMP7	19.7 (7.93–48.9)	<0.001	37.73 (11.25–126.58)	<0.001
MMP8	2.25 (1.75–2.89)	<0.001	2.07 (1.61–2.67)	<0.001
MMP9	2.46 (1.94–3.13)	<0.001	2.35 (1.84–3.01)	<0.001
MMP12	26.53 (6.54–107.61)	<0.001	28.54 (6.75–120.64)	<0.001
MMP13	1.74 (1.3–2.34)	<0.001	2.04 (1.44–2.9)	<0.001
TIMP1	0.58 (0.4–0.83)	0.003	0.58 (0.4–0.84)	0.004
TIMP2	0.25 (0.16–0.37)	<0.001	0.25 (0.16–0.38)	<0.001
TIMP3	0.84 (0.69–1.01)	0.057	0.85 (0.7–1.03)	0.096
TIMP4	3022.8 (65.04–140484.7)	<0.001	1164.4 (43.34–31287.9)	<0.001

* *Multivariate model was adjusted for age and gender*.

**Table 5:** Logistic regression model to identify the biomarkers for EPTB.

	**Univariate**		**Multivariate**	
	**OR (95% CI)**	***P***	aOR (95% CI)^*^	***P***
MMP1	0.66 (0.55–0.78)	<0.001	0.62 (0.52–0.75)	<0.001
MMP2	1.26 (1.04–1.52)	0.020	1.26 (1.04–1.53)	0.017
MMP3	1.22 (0.95–1.55)	0.115	1.21 (0.95–1.54)	0.125
MMP7	0.31 (0.21–0.45)	<0.001	0.29 (0.19–0.44)	<0.001
MMP8	0.87 (0.74–1.02)	0.084	0.84 (0.7–1)	0.052
MMP9	0.85 (0.74–0.97)	0.014	0.81 (0.7–0.94)	0.005
MMP12	0.39 (0.14–1.06)	0.064	0.37 (0.13–1.01)	0.053
MMP13	0.33 (0.17–0.66)	0.002	0.33 (0.16–0.68)	0.003
TIMP1	1.78 (1.02–3.13)	0.044	1.78 (1.01–3.14)	0.045
TIMP2	1.41 (1.15–1.73)	0.001	1.6 (1.24–2.06)	<0.001
TIMP3	1.6 (1.26–2.03)	<0.001	1.58 (1.24–2.02)	<0.001
TIMP4	0.69 (0.53–0.91)	0.008	0.65 (0.49–0.88)	0.005

* *Multivariate model was adjusted for age and gender*.

### Immunoassays

Plasma levels of human MMP-1, MMP-2, MMP-3, MMP-7, MMP-8, MMP-9, MMP-12, MMP-13 (catalog number FCSTM07-8) and human TIMP-1, TIMP-2, TIMP-3, and TIMP-4 (catalog number LKTM003) were measured using a Luminex kit purchased from R&D Systems.

### Data Analysis

The statistical significance between the various study (PTB, EPTB, LTB, and HC) population were analyzed using Kruskal-Wallis test with Dunn's multiple comparisons. Wilcoxon signed rank test were used to measure the pre-and post-treatment systemic levels of MMPs and TIMPs. ROC analysis was used to measure the specificity and sensitivity between the study groups. GraphPad Prism version 8.0 (GraphPad Software Inc., San Diego, CA) were used to perform the statistical analysis and plotting the graphs. Both multivariate (Spearman rank correlation) and principal component analysis (PCA) (non-parametric) were performed using JMP (14.0 version). Finally, regression (univariate and multivariate) analysis were performed using STATA/MP version 16.0.

## Data Availability Statement

All datasets generated for this study are included in the article/supplementary material.

## Ethics Statement

The studies involving human participants were reviewed and approved by Institutional Review Board (NIRTIEC2010007) of National Institute for Research in Tuberculosis (NIRT), Chetpet, Chennai, Tamil Nadu, India. The patients/participants provided their written informed consent to participate in this study.

## Author Contributions

GK and SB conceived and designed the experiments, analyzed the data, and wrote the paper. GK, NK, and KM performed the experiments. DB, RS, DN, VB, and SB contributed materials, reagents, analysis tools.

### Conflict of Interest

The authors declare that the research was conducted in the absence of any commercial or financial relationships that could be construed as a potential conflict of interest.
